# Cerebral Palsy among Children Visiting the Outpatient Department of Pediatric Orthopaedics in a Tertiary Care Centre

**DOI:** 10.31729/jnma.8232

**Published:** 2023-08-31

**Authors:** Ayushi Awale, Bibek Banskota, Prakash Yadav, Ganga Shakya, Ashok Kumar Banskota

**Affiliations:** 1 Hospital and Rehabilitation Center for Disabled Children, Banepa, Kavrepalanchok, Nepal; 2 Department of Orthopedics, Hospital and Rehabilitation Center for Disabled Children, Banepa, Kavrepalanchok, Nepal

**Keywords:** *cerebral palsy*, *prevalence*, *spastic diplegia*

## Abstract

**Introduction:**

Cerebral palsy is a group of neurological disorders that appear in infancy or early childhood and occur as a result of anomalies in the developing brain that impair the brain's capacity to regulate movement, maintain posture, and maintain balance. Healthcare professionals can better predict the need for the medical, rehabilitative, and support services needed by people with cerebral palsy by using accurate prevalence statistics. The aim of the study was to find out the prevalence of cerebral palsy among children visiting the Outpatient Department of Pediatric Orthopaedics in a tertiary care centre.

**Methods:**

A descriptive cross-sectional study was conducted among patients visiting the Outpatient Department of Pediatric Orthopaedics in a tertiary care centre. Data from 1 January 2018 to 31 December 2019 was collected between 25 April 2023 to 5 May 2023 from medical records after obtaining ethical approval from the Institutional Review Committee. Convenience sampling method was used. The point estimate was calculated at a 95% Confidence Interval.

**Results:**

Among 6984 children, the prevalence of cerebral palsy was 545 (7.80%) (7.17-8.43, 95% Confidence Interval). The most common type of cerebral palsy was found to be spastic diplegia 219 (40.18%).

**Conclusions:**

The prevalence of cerebral palsy among children visiting the Outpatient Department of Pediatric Orthopaedic was found to be higher than in other studies done in similar settings.

## INTRODUCTION

Cerebral palsy (CP) is a group of permanent disorders of the development of movement and posture, causing activity limitation, attributed to non-progressive disturbances in the developing foetal or infant brain.^1^ Globally, 8.1 million (7.1-9.2) or 1.2% of children under 5 years are estimated to have cerebral palsy. Over 98% reside in low-income and middle-income countries.^2^

There have been a lot of recent advances in the field of disability management worldwide, but CP still remains an inadequately addressed problem in resource-limited settings. Healthcare professionals can better predict the need for the medical, rehabilitative, and support services needed by people with CP by using accurate prevalence statistics.

The aim of the study was to find out the prevalence of cerebral palsy among children visiting the outpatient Department of Pediatric Orthopedics in a tertiary care centre.

## METHODS

A descriptive cross-sectional study was conducted among children presenting to the Outpatient Department of Pediatric Orthopedics in the Hospital and Rehabilitation Center for Disabled Children, Banepa, Kavrepalanchok, Nepal after obtaining ethical approval from the Institutional Review Committee (Reference number: B&B IRC-22-25). Data from 1 January 2018 to 31 December 2019 was collected between 25 April 2023 to 5 May 2023 from medical records. Patients <18 years of age were included in the study. Patients who had missing or incomplete data on the medical records of the hospital were excluded. Convenience sampling method was used. The sample size was calculated using the formula:


$$\begin{array}{ll} \text{n} & ={\text{Z}}^{2}\times \frac{\text{p}\times \text{q}}{{\text{e}}^{\text{2}}}\\ & =1.96^{2}\times \frac{0.50\times 0.50}{0.02^{2}}\\ & =2401 \end{array}$$

Where,

n = minimum required sample size
Z = 1.96 at 95% Confidence Interval (CI)
p = prevalence taken as 50% for maximum sample size calculation
q = 1-p
e = margin of error, 2%

The calculated sample size was 2401. On doubling the sample size, it becomes 4802. However, 6984 children were included in the study.

The data was collected from the hospital's medical records section. The necessary data were extracted from the patient's Outpatient Department notes and cerebral palsy intake form. The diagnosis of cerebral palsy was based on clinical assessment.

Cerebral palsy is classified according to the involvement of limb extremities such as monoplegia, hemiplegia, diplegia, and quadriplegia and the characteristics of the neurologic dysfunction (spastic, hypotonic, dystonic, athetotic, or a combination).^3^ Thus, the cases of cerebral palsy were distinguished into the specific type based on topography and muscle tone. The frequency of gestational age at the time of delivery (pre-term, term or post-term) among children with cerebral palsy was observed on the patient's medical records. Infants born >37 weeks are considered term, <37 weeks of gestation as preterm and >42 weeks as postterm.^4,5^

Data were entered and analyzed using IBM SPSS Statistics version 28.0. The point estimate was calculated at a 95% Confidence Interval.

## RESULTS

Among 6984 patients, cerebral palsy was seen in 545 (7.80%) (5.55-10.05, 95% CI). Out of 545 children, the most common type of cerebral palsy was spastic diplegia 219 (40.18%) (Table 1).

**Table 1:** Types of cerebral palsy (n = 545).

Cerebral palsy	n (%)
Spastic diplegia	219 (40.18)
Spastic hemiplegia (left)	54 (9.91)
Spastic hemiplegia (right)	68 (12.48)
Spastic quadriplegia	87 (15.96)
Total body involvement	35 (6.42)
Dyskinetic CP	27 (4.95)
Ataxic CP	16 (2.94)
Mixed CP	21 (3.85)
Hypotonic CP	18 (3.30)

**Figure 1:**
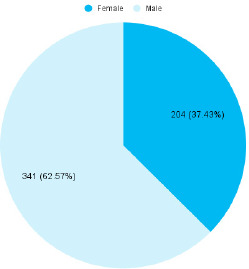
Gender-wise distribution among patients with cerebral palsy (n= 545).

Most of the children 249 (45.69%) were under the age of 5 years. Among 545 children, 341 (62.57%) were male (Figure 1).

**Figure 2:**
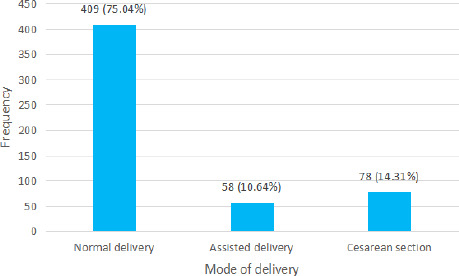
The mode of delivery among children with cerebral palsy (n= 545).

Birth asphyxia 222 (40.73%) was the most common underlying cause among children with cerebral palsy. The most common mode of delivery among the children with cerebral palsy was normal delivery 409 (75.04%) (Figure 2).

**Figure 3:**
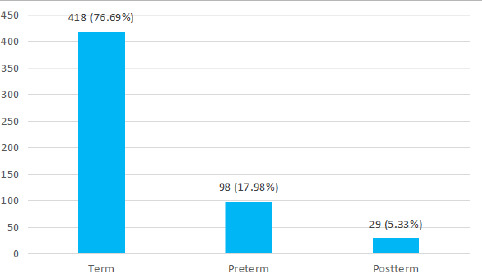
The gestational age at birth of the children diagnosed with cerebral palsy (n= 545).

Among 545 children with cerebral palsy, the commonest gestational age at delivery was term birth 418 (76.69%) (Figure 3).

## DISCUSSION

Among 6984 children, the prevalence of CP was seen at 7.80% which is lower than the study done in the same setting in Nepal in 2015 with a prevalence of 15%.^6^ About 1 in 345 children in the United States have been identified with CP, according to 2010 estimates from CDC's Autism and Developmental Disabilities Monitoring Network.^7^ It depicts that the prevalence of cerebral palsy is still high in developing countries compared to developed countries.

In our study there is a male predominance at 341 (62.57%) which is similar to another study where 64.4% of the subjects were male.^8^ Spastic diplegia was the most common type of cerebral palsy in our study which was similar to other studies.

In another study, most of the patients 74.8% had spastic CP subtype, and 54.8% had quadriplegia.^9^ The most common risk factor involved was birth asphyxia. It is similar to other studies including one that suggests that there is a significant contribution of perinatal asphyxia for cerebral palsy in normal weight term babies. In a similar study, it was confirmed that premature delivery and emergency cesarean section were significant underlying causes of cerebral palsy in children.^10^ Gestational age and small for gestational age were important predictors of cerebral palsy.^11^ In our study it was found that cerebral palsy was more common among term infants with normal mode of delivery. In contrast, the other studies identified preterm birth, and emergency cesarean section as underlying causes for cerebral palsy in children.^10^

The most common comorbidity related to cerebral palsy was epilepsy which was found to be between 18% and 35%. In our study, the prevalence of epilepsy was found to be 13.8%. In a similar study, children and young people with dyskinetic cerebral palsy had the highest prevalence of epilepsy 51.6%.^12^

The limitation of our study is the possibility of overdiagnosis of CP with the inclusion of children with tone abnormalities suggestive of CP or at risk of CP. Also, topography and severity of cerebral palsy are more difficult to ascertain in infancy, and magnetic resonance imaging and the Hammersmith Infant Neurological Examination may be helpful in assisting clinical decisions. Our study did not assess all the comorbidities by formal tests or screening questionnaires in all the children, thus missing some of the other possible co-morbidities or underreporting some of the evaluated co-morbidities. Our study is single-centered and the sample size is also small. Therefore, the results cannot be generalized in a larger population.

## CONCLUSIONS

The prevalence of cerebral palsy among children was higher than similar studies done in similar settings. Early screening and rehabilitation programs with provision for follow-up for the affected children are necessary and must be accessible to the marginalized groups in Nepal as well.
